# Limitations of Climatic Data for Inferring Species Boundaries: Insights from Speckled Rattlesnakes

**DOI:** 10.1371/journal.pone.0131435

**Published:** 2015-06-24

**Authors:** Jesse M. Meik, Jeffrey W. Streicher, A. Michelle Lawing, Oscar Flores-Villela, Matthew K. Fujita

**Affiliations:** 1 Department of Biological Sciences, Tarleton State University, Stephenville, Texas, United States of America; 2 Department of Biology, University of Texas at Arlington, Arlington, Texas, United States of America; 3 Department of Ecosystem Science and Management, Texas A&M University, College Station, Texas, United States of America; 4 Museo de Zoología, Facultad de Ciencias, Universidad Nacional Autónoma de México, Distrito Federal, México; State Natural History Museum, GERMANY

## Abstract

Phenotypes, DNA, and measures of ecological differences are widely used in species delimitation. Although rarely defined in such studies, ecological divergence is almost always approximated using multivariate climatic data associated with sets of specimens (i.e., the “climatic niche”); the justification for this approach is that species-specific climatic envelopes act as surrogates for physiological tolerances. Using identical statistical procedures, we evaluated the usefulness and validity of the climate-as-proxy assumption by comparing performance of genetic (nDNA SNPs and mitochondrial DNA), phenotypic, and climatic data for objective species delimitation in the speckled rattlesnake (*Crotalus mitchellii*) complex. Ordination and clustering patterns were largely congruent among intrinsic (heritable) traits (nDNA, mtDNA, phenotype), and discordance is explained by biological processes (e.g., ontogeny, hybridization). In contrast, climatic data did not produce biologically meaningful clusters that were congruent with any intrinsic dataset, but rather corresponded to regional differences in atmospheric circulation and climate, indicating an absence of inherent taxonomic signal in these data. Surrogating climate for physiological tolerances adds artificial weight to evidence of species boundaries, as these data are irrelevant for that purpose. Based on the evidence from congruent clustering of intrinsic datasets, we recommend that three subspecies of *C*. *mitchellii* be recognized as species: *C*. *angelensis*, *C*. *mitchellii*, and *C*. *Pyrrhus*.

## Introduction

Most character sets used for species delimitation are based on heritable traits that are intrinsic to an organism, including DNA, developmental programs, and emergent phenotypes [1–3]. Intrinsic traits are directly relevant to any lineage-based species concept because they evolve and reflect processes that occur during speciation [4–6]. However, non-heritable (i.e., extrinsic) traits are also used to delimit species. For example, multivariate climatic data (the average long-term pattern of variation in meteorological variables) corresponding to specimen localities have become popular in an integrative taxonomy framework (e.g., [7–9]). These data typically are analyzed using species distribution models (SDMs; also called climatic, ecological, or environmental niche models) and ordination methods, with inferred differences in climatic envelopes (i.e., the multivariate climatic range of a population) of putative species interpreted as evidence of evolutionary divergence in the niche. The underlying assumption of this approach is that climate must be correlated with adaptations for environmental tolerance, and therefore climatic envelopes could be used as surrogates for intrinsic eco-physiological traits [10, 11]. The validity of this practice has been assumed in many studies of species delimitation, despite the fact that data generated from climate do not result from descent with modification.

In accordance with measurement theory, a correlation between two traits is not sufficient for one to be a valid surrogate for the other. Measurements of both attributes must have a theoretical and empirical connection to the underlying phenomenon of interest (e.g., evolutionary divergence) for inferences made from surrogate measurements to be meaningful [12]. While the relevance of evolvable intrinsic traits to evolutionary divergence and speciation is unequivocal, it remains unclear whether measurements of climatic variables are appropriate proxies for intrinsic organismal traits. In the context of species delimitation, climatic data have been used almost exclusively in *post hoc* analyses to corroborate hypothesized species boundaries. While it is straightforward to incorporate any type of data into a *post hoc* analysis, yielding seemingly reasonable results, we contend that in order for data to be valid for species delimitation, those data must have inherent taxonomic signal. That is, an extrinsic trait dataset should minimally be able to delineate biologically meaningful entities when subjected to the same objective, operational inference procedures commonly applied to intrinsic datasets (i.e., evolutionary or statistical models that algorithmically delineate groups).

Geographically structured (i.e., taxonomically relevant) variation is well established for genetic and phenotypic data (for example, consider the main premise of phylogeography); however, the geographic clustering of climatic data associated with specimen localities and its congruence with groups inferred from intrinsic datasets has not been explored previously in species delimitation studies. In this study we used ordination (principal components analysis; PCA) followed by model-based clustering [13, 14] to detect optimal number and assignment of specimens to putative species within the speckled rattlesnake (*Crotalus mitchellii*), a pitviper inhabiting the Mohave and Sonoran deserts of western North America [15]. Specifically, we examined congruence of clusters obtained from climatic data to those inferred using mtDNA, nDNA, and phenotypic datasets, in order to determine whether climatic data exhibit taxonomic signal that is similar to that of these intrinsic trait datasets.

In contrast to genetic or phenotypic traits, which in sympatry show variation between individuals and species, climate for a given location and spatial scale is always singular at a given point in time (i.e., a locality cannot simultaneously be both mesic or xeric, temperate or tropical). Therefore, ordination or clustering algorithms applied to climatic layers corresponding to geographic localities (hereafter termed climate clusters) will be delineated based on variation in temperature and precipitation of a study region rather than on the genetic and phenotypic variation of any particular group of organisms inhabiting that region. Thus, distinct climate clusters should exhibit little or no spatial overlap, and any particular cluster may form a mosaic of disjunct patches across space depending on the extent of the study area. In contrast, because biological variation is often complex over a given spatial extent, clusters delineated from intrinsic traits may overlap spatially, but separate groups should generally be geographically contiguous owing to the homogenizing influence of gene flow within sexual species. The fundamental differences in the properties of intrinsic and extrinsic data may lead to fundamentally different patterns when analyzed using the same statistical algorithms, such that the two types of data do not have similar relevance to questions concerning species delimitation.

**Fig 1:**
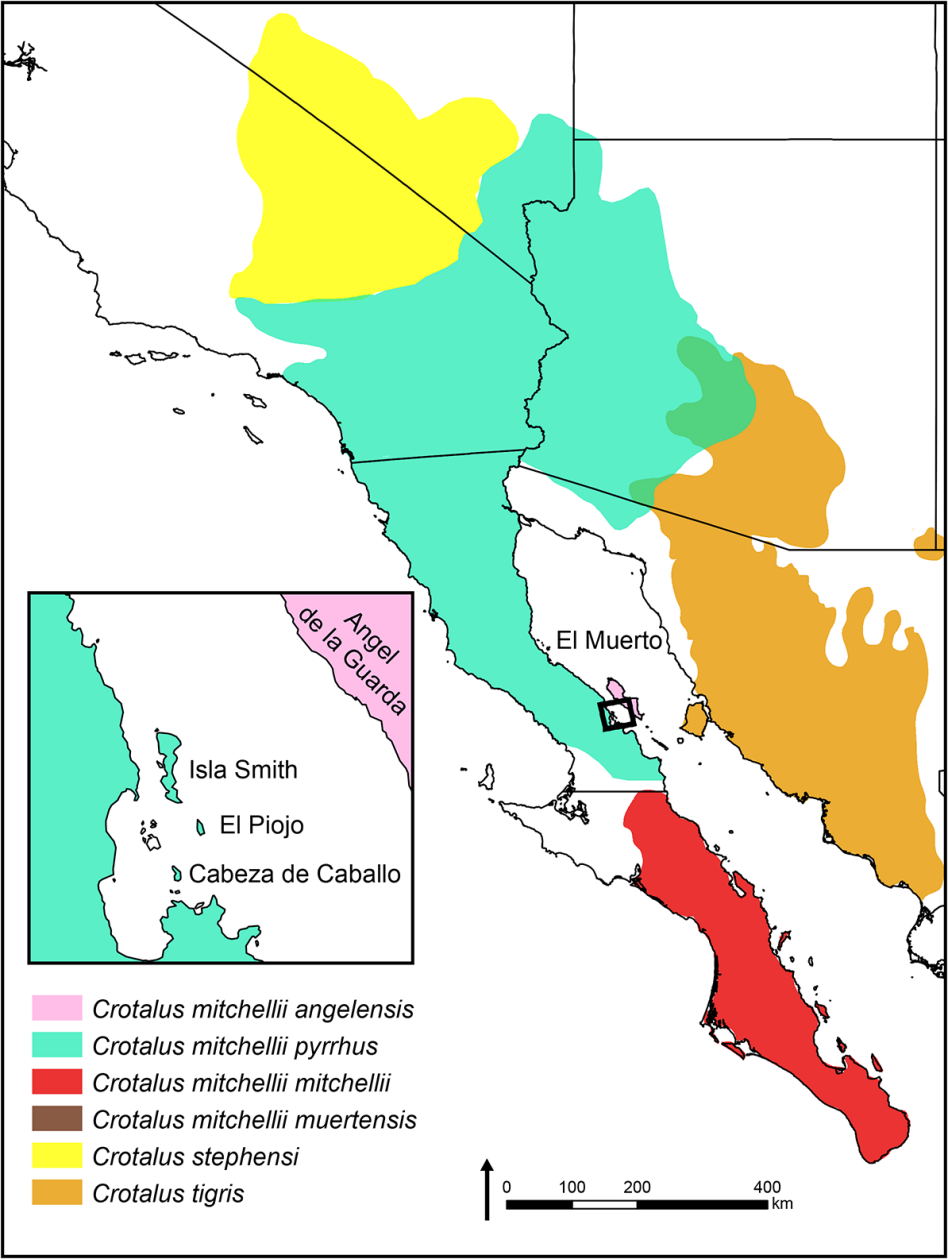
Geographic distribution of the *Crotalus mitchellii* group in western North America, including currently recognized subspecies of *C*. *mitchellii* (based after [15]). Inset shows islands in the Los Angeles Bay region of the central Gulf of California that are inhabited by *C*. *mitchellii*, *sensu lato*.

Currently, three species, *C*. *mitchellii*, *C*. *tigris*, and *C*. *stephensi*, constitute the *C*. *mitchellii* species group (sensu [16]); however, in recent molecular studies *C*. *tigris* has not been placed as sister to *C*. *mitchellii* [17], and *C*. *stephensi* has yet to be included in a large-scale analysis of rattlesnake relationships. Speckled rattlesnakes are highly variable in color pattern, head scalation, venom properties, and morphometric proportions, which may have obfuscated species-level diversity [15, 18–20]. Two primarily mainland subspecies (*C*. *m*. *mitchellii* from the southern half of the Baja California Peninsula, and *C*. *m*. *pyrrhus* from the northern half of the peninsula, and extending into the southwestern United States) and two insular subspecies (*C*. *m*. *muertensis* from El Muerto Island, and *C*. *m*. *angelensis* from Ángel de la Guarda Island, both in the Gulf of California) are currently recognized ([15]; Fig 1). Although the taxonomic status of the insular subspecies has been subject to speculation (e.g., [15, 21]), no modern studies have included the formal evaluation of data. Several populations of speckled rattlesnakes occur on additional islands off the coast of the Baja California Peninsula. These insular populations are known from few specimens and are assumed to represent the nearest mainland subspecies, but their taxonomic status has never been evaluated. Although natural history and distribution is comparatively well known for *C*. *mitchellii*, taxonomy is outdated and only partially resolved, as recent studies included few individuals from Mexico, the apparent center of phenotypic and genetic diversity for this complex [18]. In addition to samples from throughout the mainland distribution of the group, for this study we included phenotypic data from all island populations and genetic data from specimens collected from El Muerto, Smith, Piojo, Cabeza de Caballo, Ángel de la Guarda, and Monserrate islands in the Gulf of California.

## Results

### Ordination and Clustering of Genetic Data

**Fig 2:**
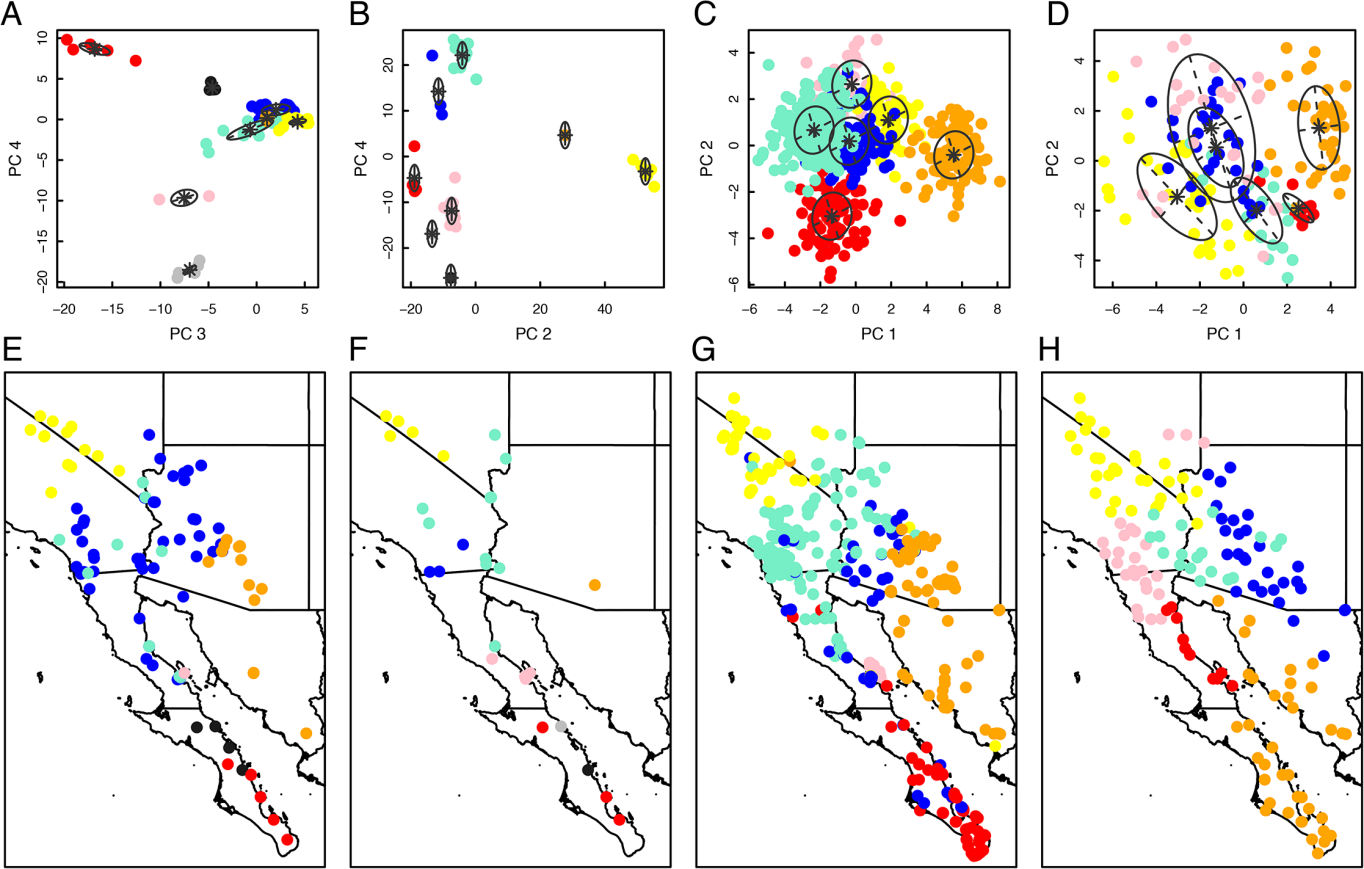
Ordination (PCA) and clusters of mtDNA sequence (*A*), nDNA SNP (*B*), phenotypic (*C*), and climatic (*D*) datasets in geographic space, (*E*,*F*,*G*,*H*, respectively). Clusters are based on PC axes that explained > 5% of total variation for each analysis (excluding axis 1 for nDNA, which was correlated with amount of missing data). Choice of PC axes presented on scatterplots is arbitrary (e.g., PC3 and PC4 are presented in *A* because the “grey” cluster, which represents specimens from Cabeza de Caballo Island, is overlaid with other clusters and is not visible using other axes); see S2 Fig for scatterplots of additional PC axes.

**Fig 3:**
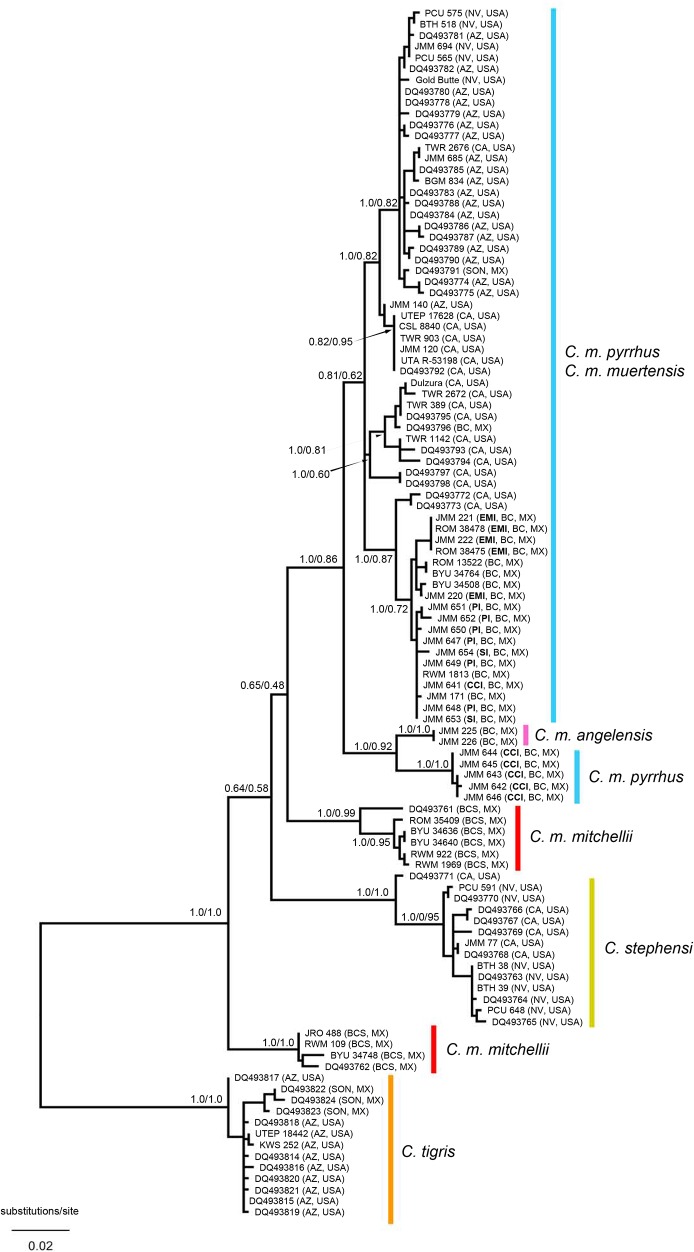
Genealogy of mitochondrial ATPase 8 and 6 genes for 108 individuals of the *Crotalus mitchellii* group (including all recognized taxa). Internodes are labeled with posterior probabalities/ML bootstrap support values. PI = Piojo Island, CCI = Cabeza de Caballo Island, SI = Smith Island, EMI = El Muerto Island.

PCA of 590 bp of mtDNA ATPase 8 and 6 genes followed by model-based clustering using mclust for 108 specimens of the *C*. *mitchellii* group (including 13 *C*. *tigris* and 14 *C*. *stephensi* samples) yielded eight clusters that were largely geographically contiguous and mostly corresponded to previously recognized taxonomic groups (Fig 2A and 2E). As expected, *C*. *stephensi* and *C*. *tigris* were each inferred as discrete clusters. Within the *C*. *mitchellii* complex, discrete clusters corresponded to each of two populations on Ángel de la Guarda and Cabeza de Caballo islands in the Gulf of California; however, samples from El Muerto Island (*C*. *m*. *muertensis*), also in the Gulf of California, clustered predominately with mainland samples of *C*. *m*. *pyrrhus*, as did the remaining island populations from the region. Two geographically contiguous groups from Baja California Sur corresponded to *C*. *m*. *mitchellii*. Two overlapping, and mostly geographically contiguous, groups extended from southern Nevada to the mid-peninsular region, and corresponded to *C*. *m*. *pyrrhus*. These clusters were not strongly separated by ordination and individuals exhibited high levels of classification uncertainty (S1 Fig); thus, we interpret these two clusters as representing a single species with high mitochondrial diversity. Phylogenetic analyses using Bayesian and maximum likelihood tree-searching criteria inferred clades corresponding to each of these clusters; however, the two *C*. *m*. *mitchellii* clades are not sister groups, and the endemic haplogroup from Cabeza de Caballo Island renders *C*. *m*. *pyrrhus* paraphyletic (Fig 3).

PCA of 1826 nDNA SNPs followed by model-based clustering for 41 specimens of the *C*. *mitchellii* group (including one *C*. *tigris* and five *C*. *stephensi* samples) also yielded eight clusters (Fig 2B). These clusters were mostly geographically contiguous (Fig 2F) and many were congruent with patterns from mtDNA (including *C*. *m*. *muertensis*, which clustered with mainland *C*. *m*. *pyrrhus*); only some clusters from the central and southern parts of the Baja California peninsula were discordant with those inferred from mtDNA. Three SNP clusters corresponded to specimens in the southern Baja California peninsula (*C*. *m*. *mitchellii*), but two of these clusters consisted of singletons with considerable missing data. Although these clusters may represent deep nuclear diversity, based on phylogenetic analyses and Bayesian clustering using STRUCTURE (see below), we consider it more likely that the clustering pattern is an artifact of missing data in the SNP dataset for these individuals. Whereas specimens from the central Baja California peninsula were partitioned into three different mitochondrial clusters (mainland *C*. *m*. *pyrrhus*, including Smith and Piojo islands; Cabeza de Caballo Island; and Ángel de la Guarda Island), SNP data for all island samples from the central peninsula region (Ángel de la Guarda, Smith, Piojo, and Cabeza de Caballo islands) grouped together with specimens from the adjacent mainland.

**Fig 4:**
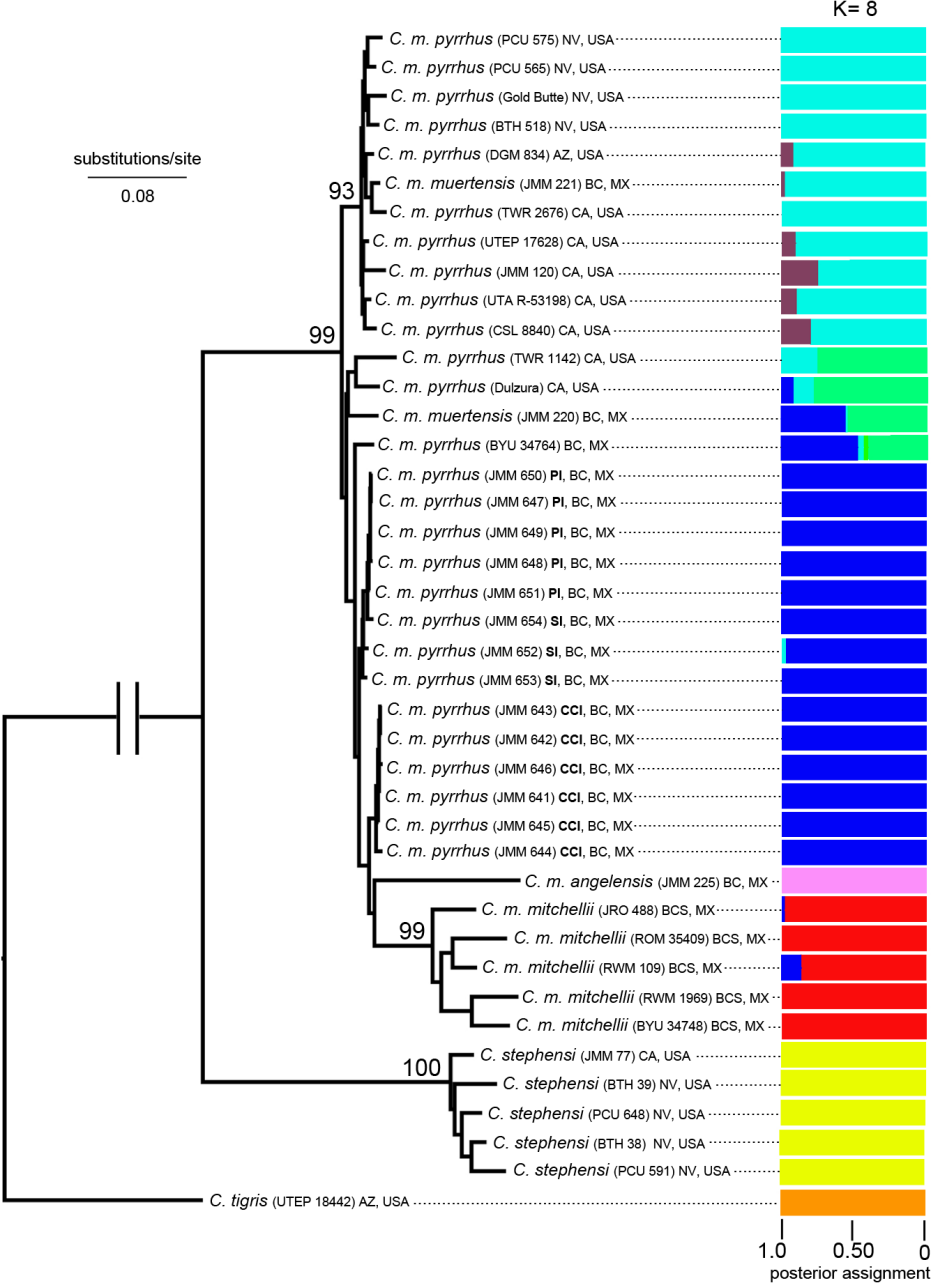
nDNA SNP genealogy for the *Crotalus mitchellii* species complex, with posterior assignment of each individual to clusters using STRUCTURE. The SNP genealogy is based on maximum likelihood analysis of 2318 concatenated SNPs; numbers indicate boostrap support for major clades. The STRUCTURE analysis is based on 1826 biallelic SNPs. PI = Piojo Island; SI = Smith Island; CCI = Cabeza de Caballo Island.

To further investigate discrepancies between mtDNA and nDNA datasets in the central peninsula region, we performed a phylogenetic analysis of the concatenated alignment of 2318 genome-wide SNPs using maximum likelihood, and also implemented Bayesian clustering of 1826 SNPs using the program STRUCTURE 2.3 (Fig 4). STUCTURE uses genotypic data to probabilistically assign individuals to either discrete populations or jointly to two or more populations when allelic variation is consistent with genomic admixture [22]. In contrast, mclust uses Expectation-Maximization (EM) to identify distance-based clusters with Gaussian mixture models and BIC [23]. Discrepancies in the number of SNPs between analyses reflect a tradeoff to maximize samples for the STRUCTURE analysis (which incorporates heterozygous SNPs), and to maximize number of SNPs per sample for the phylogenetic analysis (at the expense of excluding specimens with over 20% missing data). The ML phylogeny indicated a weakly supported series of branches for this region: Smith + Piojo, and then Cabeza de Caballo, as successively sister to a clade consisting of *C*. *angelensis* + *C*. *m*. *mitchellii*. All these internodes are extremely short; however, the branch leading to the specimen from Ángel de la Guarda is long and support for a single *C*. *m*. *mitchellii* clade is high. Rather than grouping all island populations from the central peninsula region into a single cluster, the STRUCTURE analysis inferred two clusters with high posterior assignment of specimens: one cluster consisted of specimens from Smith, Piojo, and Cabeza de Caballo islands, while the other consisted of the specimen from Ángel de la Guarda Island. Finally, the STRUCTURE analysis inferred a single cluster for all specimens from the southern half of the Baja California peninsula corresponding to *C*. *m*. *mitchellii*.

### Ordination and Clustering of Phenotypic Data

PCA of 27 quantitative phenotypic traits followed by model-based clustering for 576 specimens from the *C*. *mitchellii* group (including 91 *C*. *tigris* and 54 *C*. *stephensi* specimens) inferred six clusters that were largely geographically contiguous and mostly corresponded to previously recognized taxonomic groups (Fig 2C and 2G). These groups corresponded closely to *C*. *tigris*, *C*. *stephensi*, *C*. *m*. *mitchellii*, *C*. *m*. *angelensis*, and *C*. *m*. *pyrrhus* (but not *C*. *m*. *muertensis*). A sixth group (depicted in blue in Fig 2C and 2G) showed only moderate geographic contiguity, relatively high uncertainty of classification, and overlapped with groups corresponding to both *C*. *m*. *mitchellii* and *C*. *m*. *pyrrhus*, including many individuals from islands (particularly Piojo and Cabeza de Caballo islands). Specimens assigned to this group were either subadults or relatively small adults, and indicated geographic variation in body size (e.g., insular dwarfism). Thus, we consider this sixth group to be an ontogeny-influenced cluster.

### Ordination and Clustering of Climatic Data

PCA of 19 climatic variables followed by model-based clustering for localities corresponding to the 576 specimens from the *C*. *mitchellii* group for which we had phenotypic data (including 91 *C*. *tigris* and 54 *C*. *stephensi*) inferred six clusters (Fig 2D and 2H). Although most were geographically contiguous, climate clusters did not correspond to any groups inferred using other datasets or to any previously recognized taxonomic units; rather climate clusters delineated regions with distinctive temperature and rainfall regimes (e.g., the cluster depicted by orange symbols in Fig 2H corresponds with a subtropical region with strong monsoon influence). Examination of PC factor loadings indicates that the first component axis is structured largely by a gradient in temperature, and the second by a gradient in precipitation (S1 Table). In general, localities from different clusters showed little spatial overlap. Distributions of some taxonomic groups identified from congruence of intrinsic datasets (*e*.*g*., *C*. *m*. *mitchellii* and *C*. *m*. *angelensis*) encompassed only small regions within climate clusters with larger spatial extents, whereas distributions of other groups (e.g., *C*. *m*. *pyrrhus*, *C*. *tigris*) overlapped with the spatial extents of multiple climate clusters.

### Tests of Introgression

Given some incongruent patterns in our mtDNA and nDNA analyses for specimens from the central Baja California peninsula and associated islands, we tested for the signature of introgressive hybridization using four taxon Patterson’s D-statistics [24]. The results of these tests indicated two instances of historical introgression that occurred within the *C*. *mitchellii* group: (*i*) between *C*. *m*. *pyrrhus* and *C*. *stephensi*, and (*ii*) between *C*. *m*. *angelensis* and *C*. *m*. *pyrrhus* populations from islands in Los Angeles Bay (Smith, Piojo, and Cabeza de Caballo islands) and the adjacent peninsular mainland (S2 Table).

### Species Limits and Coalescent Species Tree Analysis

**Fig 5:**
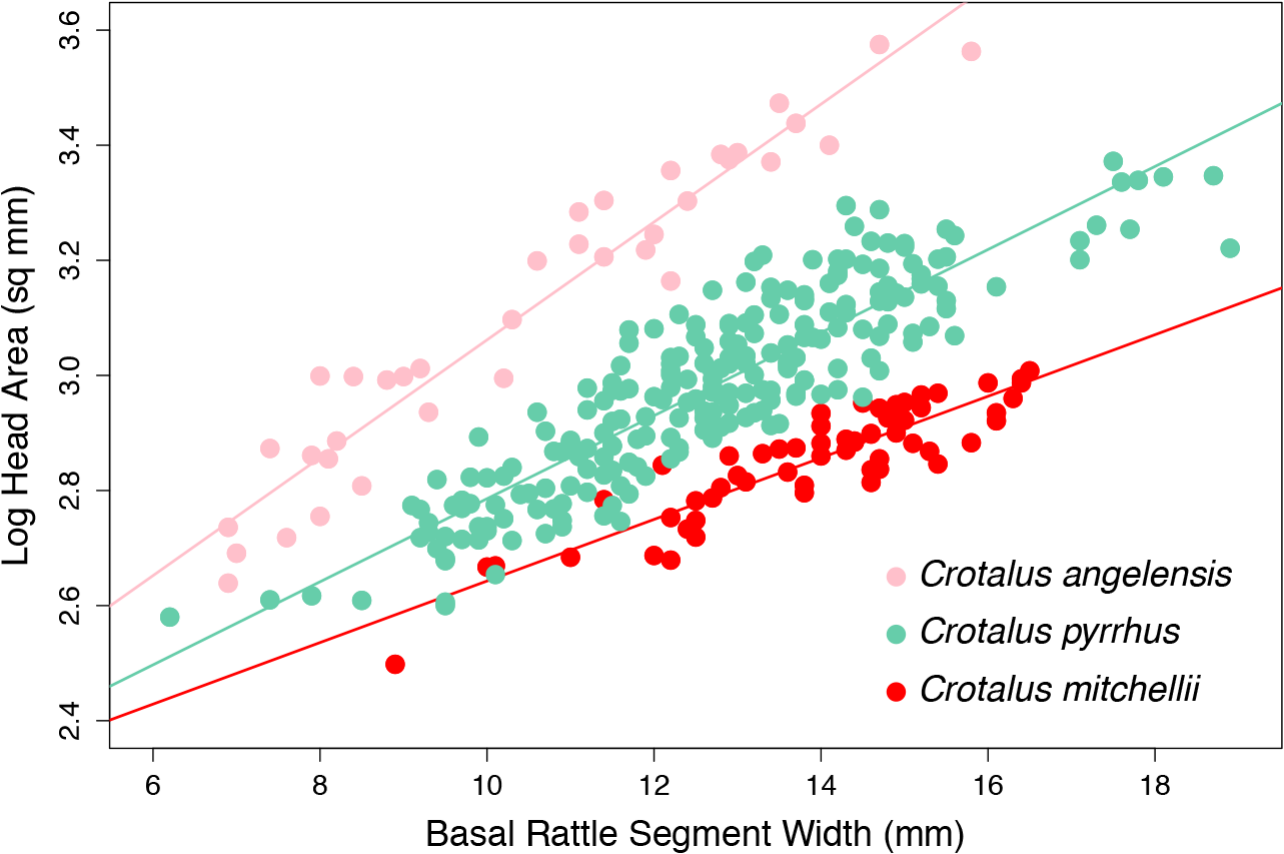
Allometric scaling of head size as a function of rattle size among the species of the *Crotalus mitchellii* complex. Each species demonstrates distinct differences in developmental trajectories (ANCOVA homogeneity of slopes test; *df* = 2,321, *F* = 536.73, *P* < 0.0001).

Based on congruent clustering patterns using mtDNA, nDNA, and phenotypic data, we propose that the currently recognized subspecies *C*. *m*. *mitchellii*, *C*. *m*. *angelensis*, and *C*. *m*. *pyrrhus* be recognized as species. The insular population *C*. *m*. *muertensis* (recognized as *C*. *muertensis* by Grismer [21]) exhibits little or no differentiation from mainland populations of *C*. *pyrrhus*, and we therefore recommend that this population be considered part of *C*. *pyrrhus*. The widespread taxon *C*. *pyrrhus* comprises at least two partially overlapping clusters each for mtDNA, nDNA, and phenotypic data, reflecting relatively high diversity in this species, and additional insular populations currently recognized as *C*. *pyrrhus* may warrant species recognition (e.g., the population from Cabeza de Caballo Island has a distinctive mitochondrial haplogroup, and additional insular populations also show considerable phenotypic divergence). However, we refrain from making further taxonomic decisions pending additional analysis, as the evolutionary dynamics of insular speckled rattlesnakes appear to be complex. Although *C*. *mitchellii* includes two deep and geographically contiguous mitochondrial clades, the SNP phylogeny, STRUCTURE analysis, Patterson’s D statistic analysis, and phenotypic clustering all support a single species. The three species of the *C*. *mitchellii* complex proposed herein are readily distinguishable by differences in allometric scaling of head size and rattle size (Fig 5). Furthermore, previous studies have noted that *C*. *mitchellii* has considerably more toxic venom and shorter fangs than does *C*. *pyrrhus* [25, 26].

**Fig 6:**
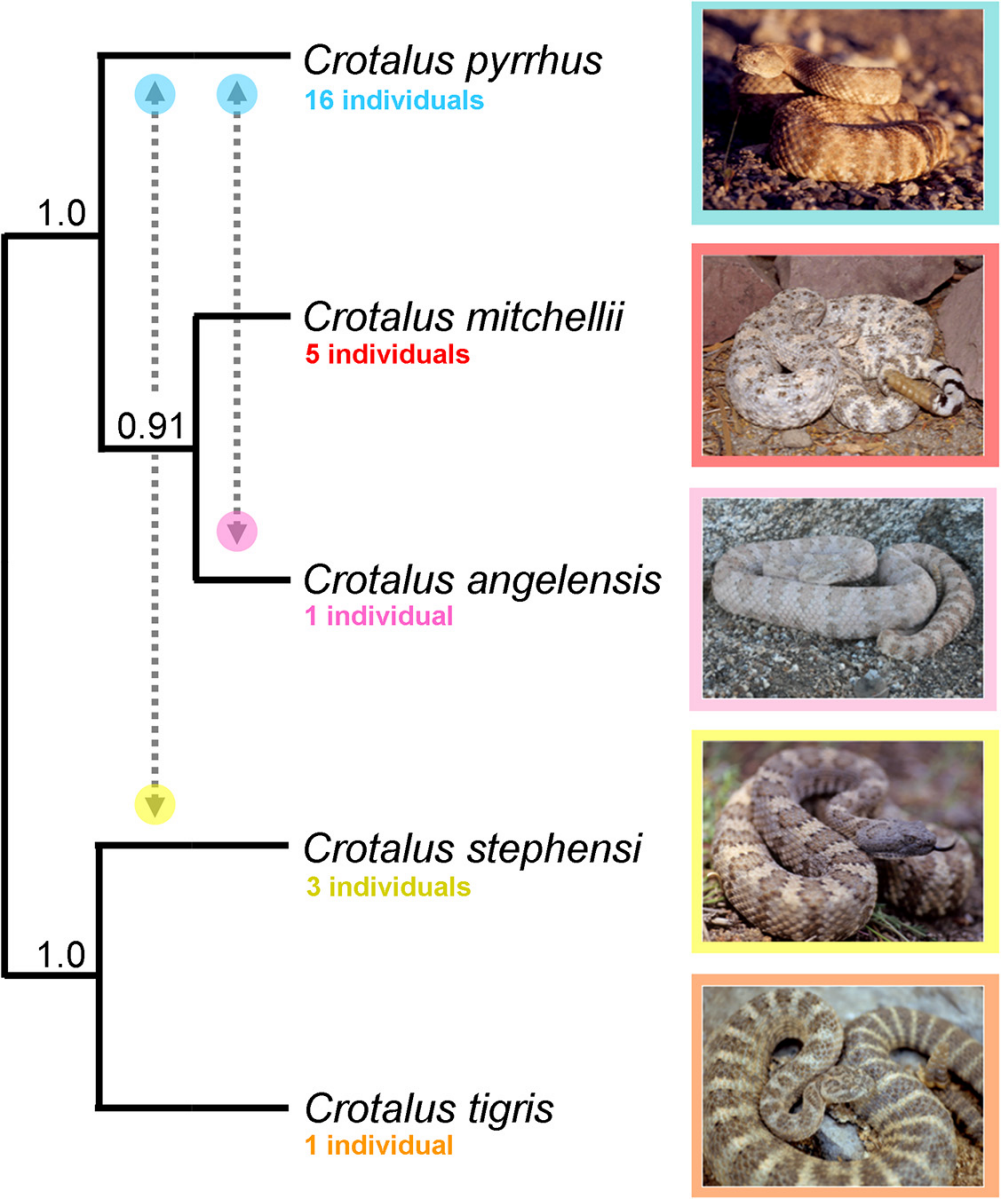
Species tree for species recognized within the *Crotalus mitchellii* group. The species tree was assembled using a full coalescent model applied to 2409 biallelic SNPs; support values represent proportion of trees sampled with a particular topology. Dashed lines with arrows indicate taxa for which evidence of introgression was found using Patterson’s D-statistics. Photograph of *C*. *mitchellii* by M. Ingrasci.

We designated a five-species taxonomy (*C*. *tigris*, *C*. *stephensi*, *C*. *mitchellii*, *C*. *pyrrhus*, and *C*. *angelensis*) for coalescent species tree analysis of 2409 biallelic SNPs. The analysis inferred a monophyletic *C*. *mitchellii* complex with *C*. *angelensis* and *C*. *mitchellii* as sister species (Fig 6). *Crotalus stephensi* and *C*. *tigris* were also supported as sister species.

## Discussion

### Mito-nuclear Discordance

Although clustering patterns were congruent for purposes of species delimitation, mitochondrial and nuclear datasets were discordant in estimates of phylogenetic relationships at deep levels in the phylogeny for *C*. *mitchellii* as well as for insular populations in the central Baja California region corresponding to *C*. *pyrrhus* and *C*. *angelensis*. In *C*. *mitchellii*, deep coalescence results in two divergent mitochondrial clades that render this taxon non-monophyletic with respect to *C*. *pyrrhus* + *C*. *angelensis*, and possibly *C*. *stephensi* (Fig 3); however, the species was inferred as monophyletic from the ML analysis of nuclear SNP data and comprised a single phenotypic cluster (Fig 4). These observations suggest that past barriers to gene flow between deep mitochondrial clades of *C*. *mitchellii* were either porous or transient enough not to affect strongly the nuclear genome. Deep mitochondrial subdivisions may develop in species with low vagility in the absence of complete barriers to gene flow [27, 28]; thus, a specific vicariant event need not be invoked. Furthermore, the long and narrow peninsula of Baja California may enhance demographic effects on mitochondrial phylogeographic structure by linearizing the geographic arrangement of metapopulations, which would restrict zones of contact, and hence gene flow, between subpopulations (in general, terrestrial vertebrates in peninsular Baja California have extensive phylogeographic structure in mitochondria [29, 30]).

Rattlesnake populations from the land-bridge islands of Los Angeles Bay (Smith, Piojo, and Cabeza de Caballo) and the large deep-water island of Ángel de la Guarda (~12 km northeast of Los Angeles Bay across the Ballenas Channel) also exhibit mito-nuclear discordance. Ángel de la Guarda is an ancient island (~2 my [31]), on which the rattlesnake population has had sufficient time to diverge from the mainland. In contrast, the islands in Los Angeles Bay shared a connection with the peninsular mainland within the past 10 ky [32], and should be closely related to mainland samples of *C*. *pyrrhus*. In general, this is the pattern we inferred from mitochondrial DNA, except for the population from Cabeza de Caballo Island, which forms a deeply divergent clade sister to *C*. *angelensis*. However, the ML analysis of nuclear SNP data indicates that weakly supported branches leading to populations in Los Angeles Bay and Ángel de la Guarda may render *C*. *pyrrhus* paraphyletic with respect to *C*. *mitchellii* (Fig 4).

Oversea dispersal by speckled rattlesnakes has been confirmed by molecular data directly (a single sample from Cabeza de Caballo had a mainland mtDNA haplotype, but an island-endemic nuclear genome), and by the sister-group relationship between the populations on Ángel de la Guarda and Cabeza de Caballo islands, which have never shared a terrestrial connection. Our D-statistic analyses indicate that periodic trans-marine dispersal and introgression from Ángel de la Guarda into the islands of Los Angeles Bay (and likely the peninsular mainland) best explains the discordant patterns observed between mtDNA and nDNA data. This dispersal scenario is also supported by prevailing surface currents in the Gulf of California, which form a counterclockwise gyre moving toward the south and west along the Baja California coast during spring and summer active seasons [32]. Oversea dispersal may be a consequence of the great tidal amplitudes in the central Gulf (> 4m during spring tides; [29]) coupled with the apparent proclivity of insular speckled rattlesnakes for foraging in intertidal zones ([33], JMM personal observation). It is plausible that snakes seek refuge in crevices and rock cavities in intertidal zones during low tides, and are then occasionally swept out to sea as refugia become inundated during high tides. Such events can explain the episodic influx of genes from Ángel de la Guarda Island rattlesnakes to populations on islands in Los Angeles Bay and the adjacent peninsular mainland.

### Climatic Data and Species Delimitation

The justification for using climatic data in species delimitation is based on the idea that distinct species occupy distinct ecological niches, a widespread interpretation of Van Valen’s [34] proposals concerning the nature of species. We do not deny that ecological traits may play an important role in speciation; however, our results from ordination and model-based clustering of multiple datasets demonstrated that climatic data do not have the inherent taxonomic signal that is evident with genetic and phenotypic data, calling into question the use of climatic data in species delimitation. The only previous application of climatic data to species delimitation methods that algorithmically delineate groups likewise did not result in the discovery of meaningful clusters, in this instance based on congruence of assignment with traditional taxonomic criteria [35]; however, these authors did not question the validity of using climatic data for delimiting species. While the reason for these conflicting results is straightforward, i.e., climate develops and changes through non-evolutionary mechanisms, the implications are underappreciated for practical systematics. Our argument is simple: (*i*) climate is generated by non-evolutionary processes; (*ii*), ordination and clustering of climatic data will not delineate biological entities, but rather prevailing precipitation and temperature regimes; (*iii*) thus, climatic data do not possess inherent taxonomic signal; (*iv*) therefore, climatic data are irrelevant for delimiting species entities using ordination and clustering methods. If these propositions are correct, then the interpretation of divergence in underlying eco-physiological traits does not follow from the ordination of climatic data, even though divergence in ecological traits may be reflected in some climate clusters.

Particularly noteworthy was the failure of the climatic dataset to identify the deeply divergent population from Ángel de la Guarda Island, whereas all other datasets unambiguously delineated this population as a distinct group. *Crotalus angelensis* apparently has been evolving in isolation since Ángel de la Guarda Island was sheared from the peninsular mainland approximately 2 mya by tectonic rifting [31], but this vicariant event is intractable on the basis of climate, because the island lies in an exceptionally arid and relatively uniform climatic zone that includes most of the northeastern peninsula and its nearshore islands. Most of the six climate clusters spanned multiple species boundaries and attest to the problems with delineating species boundaries based on climate. For example, the distribution of *C*. *pyrrhus* as delineated by intrinsic trait data encompasses all six climate groups, at least marginally, while *C*. *mitchellii* and *C*. *angelensis* are found exclusively within subregions of single climate clusters. This is not to say that climate clusters are biologically uninformative. For example, the parapatric boundary between *C*. *mitchellii* and *C*. *pyrrhus* in the midpeninsular region is the only species boundary that also corresponds to a boundary between climate clusters, and indicates a possible role of climate in the divergence of these species, or in the current maintenance of species boundaries. Additionally, the application of distribution modeling techniques may provide important information on niche similarity among closely related species, or on which climatic variables best explain distributions of different species, and could thereby indicate ecological axes along which divergence may have occurred [36]. However, we emphasize that this information is useful only in a post-species delimitation context and does not provide additional evidence for species boundaries. For example, simply because the boundary between *C*. *mitchellii* and *C*. *pyrrhus* coincides with a shift in climate, the evidence for species boundaries is not stronger for this pair of taxa than it is for the boundary between *C*. *tigris* and *C*. *pyrrhus*, which does not correspond with a distinctive shift in climate.

In addition to the issue of taxonomic signal as it pertains to species delimitation and assignment, there is a broader issue of the general relationship between intrinsic physiological tolerance and climate. Although a species obviously cannot maintain populations outside a specific range of climatic conditions, it remains unclear theoretically what type of object is modeled by the most frequently used set of climatic variables (derivatives of temperature and precipitation), and its connection to underlying intrinsic traits of the organisms with which they are associated [37]. When treated in aggregate (i.e., in a multivariate space) the use of climatic data as a surrogate for an intrinsic biological trait seems intuitive, with population- and species-specific means, variances, and other parameters that could be plotted and conceptualized as environmental tolerance. But when considered at the level of individual climatic variables, for example “Mean Temperature of Driest Quarter,” they are nonsensical as proxies for such traits. Climatic envelopes should be interpreted as a multivariate property of the environment of species, rather than as an intrinsic trait of an organism or species, or as a proxy for such a trait. Environmental tolerance arises from myriad intrinsic and extrinsic properties that vary in importance from species to species, and range from enzymatic structure and function, to behavioral thermoregulation, to evaporative transpiration. A multitude of different traits may be involved, which could result in the same outcome regarding environmental tolerance as represented by different species’ climatic envelopes, none of which is distinguishable using climatic data. Thus, measurements generated from climatic data differ from many other emergent properties of populations, such as summary statistics of allele frequencies, because there is no direct homology between intrinsic properties of organisms and climate.

Because climate is a property of locations rather than of organisms, we contend that climatic variables do not reflect attributes of evolving genetic and phenotypic systems, and are therefore invalid surrogates for intrinsic eco-physiological traits. Although this proposition may seem obvious, the frequent use of climatic data to infer evolutionary divergence in species delimitation has prompted us to use a *reductio ad absurdum* argument with an empirical example to demonstrate that climate clusters do not possess inherent taxonomic signal. Our study focused specifically on clustering and ordination rather than on correlative SDMs *per se*, and while these methods involve different analytical procedures, in context of species delimitation both make assertions of evolutionary divergence from patterns based on climatic data exclusively, generally without mention of alternative explanations (for example, statistical differences in climatic envelopes between parapatric or allopatric species can be explained simply by the geography of climate rather than through divergence in intrinsic traits [38, 39]). However, most studies that use climatic data for species delimitation actually incorporate these datasets only after candidate species have been identified and specimens assigned by other means. Correlative SDMs are phenomenological regression models, and require that individual locality records be correctly assigned to species *a priori* for results to have interpretive validity (i.e., model structure and functional forms are dependent on parameterization of models with data; these models perform poorly when distribution and taxonomy are uncertain or incomplete [40, 41]). Insofar as species delimitation means to define or establish hypotheses of species boundaries, the use of SDMs to delimit or validate species involves circular reasoning; therefore, in practice these analyses are used mostly to describe the climatic envelopes of species. However, it is clear from previous arguments provided by researchers of species delimitation that in general climatic data (used either in ordination or in SDMs) are incorrectly perceived as providing evidence for species boundaries [7–9, 42, 43].

## Materials and Methods

### Species Delimitation

Speciation is a temporally extended process that involves initially semi-permeable barriers to gene flow as genetic differences with negative fitness effects accumulate between nascent lineages [5]. In accordance with this view, we consider any data that reflect aspects of divergence and speciation to be suitable criteria for species delimitation [6]. For this study, we consider the most inclusive of congruent clusters inferred from two or more data sources that also exhibit geographic contiguity to be a conservative estimate of species boundaries. In addition, optimal cluster assignments may include multiple non-discrete groups that reflect high diversity within a single species; thus, we also considered ordination plots and whether geographically overlaid clusters showed high classification uncertainty.

### Genetic Data

Collecting permits for specimens obtained for this study were issued by the government of Mexico (SEMARNAT) to OFV (04769/09, 07266/12), and by the state of Arizona to JMM (M30207246). Research was carried out under University of Texas at Arlington IACUC no. A07.027. We generated sequence data for mitochondrial ATPase 8 and 6 loci (590 bp) for 56 specimens from the *C*. *mitchellii* group (S3 Table), and augmented these sequences with those from a previous study [18] for a total of 108 specimens from throughout the geographic distribution (including several insular populations from the Gulf of California). Tissues were obtained from scale clips, muscle, liver, or blood. We PCR amplified and sequenced fragments using previously described laboratory protocols [44]. We used double-digest restriction site-associated DNA sequences (ddRADseq) to sample SNPs from throughout the nuclear genome [45]. We generated SNPs from a subset of 41 individuals from the mtDNA analysis, including samples from each supported mtDNA clade (corresponding to *C*. *tigris*, *C*. *stephensi*, and all mtDNA clades within the *C*. *mitchellii* complex). We digested genomic DNA using SbfI and MspI restriction enzymes and constructed ddRADseq libraries using New England Biolabs (NEB) reagents, as described in [46]. Size selection of libraries was performed on the Blue Pippen platform (Sage Sciences, Beverly, MA). A single lane of PE100 high-throughput sequencing was conducted at the UT Southwestern genomics facility using an Illumina Hi-Seq 2100.

### Phenotypic Data

We collected external phenotypic data from ethanol-preserved specimens of *C*. *mitchellii* and the presumably closely related species *C*. *tigris* and *C*. *stephensi* (*C*. *mitchellii* group) housed in various natural history repositories in the United States and Mexico. Although our sample included specimens from each species of the *C*. *mitchellii* group, we focused primarily on the *C*. *mitchellii* complex [20], including all insular populations. We compiled a detailed dataset for 576 specimens examined (S1 File), all of which were georeferenced to locality of origin. Adult and sub-adult specimens were selected on the basis of locality data availability and whether condition of specimens was suitable for accurately recording all variables. We estimated ontogeny by examining the rattle structure for equal width of successive rattle segments, indicating that growth was asymptotic at the time of preservation [47]. Sex was determined by evaluating presence of hemipenes. For each specimen we collected data on a series of 18 meristic (e.g., counts of scale series and body pattern elements) and nine morphometric (linear measurements) characters. For bilateral scale characters, we used the average count from each side in all statistical procedures. All characters and abbreviations are defined in S1 File.

### Climatic Data

Nineteen climatic variables corresponding to the collection localities of all specimens examined for phenotypic data were obtained from WorldClim [48] at a 2.5 arc-minute resolution. These variables are derived from extrapolations of the means and extremes of monthly, quarterly, and annual temperature and precipitation measurements [49]. We used all 19 variables because western North America is a heterogeneous environment and local populations may respond differently to particular climatic variables.

### Ordination and Clustering

We used model-based clustering to assign specimens (and locality records) to the optimal number of discrete groups without *a priori* assumptions of species assignment for mtDNA, nDNA, phenotypic, and climatic datasets. Before performing cluster analyses, we reduced the dimensionality of each dataset by PCA using correlation matrices, and retained all individual components that accounted for greater than 5% of the total variance. Morphometric variables were log_10_-transformed and regressed against log_10_-body length; residuals were retained for PCA and body length was excluded. Climatic variables related to precipitation were log_10_-transformed for normality. We applied iterative Expectation-Maximization (EM) methods and Gaussian mixture models (GMM) to identify optimal arrangements of clusters that best fit the structure of our reduced-dimension datasets [23]. Gaussian mixture models are advantageous over traditional *K*-means clustering because they allow comparisons of clusters with different volumes, orientations, and shapes (e.g., spherical, elliptical), which may more accurately reflect divergence between groups [13, 23]. Support for competing models was assessed through the Bayesian Information Criterion (BIC), which balances information explained with number of parameters used in a particular model. For the climatic dataset, we accounted for spatial sampling bias in occurrence records [50] by overlaying a grid of 0.5 minutes of a degree over the spatial extent of combined specimen occurrences and then randomly sampling one occurrence within each grid cell. We then applied the iterative EM and GMM to identify optimal arrangements of clusters for the subsample and used BIC to choose the best model. We repeated this analysis 1000 times to delimit optimal number and assignment of clusters. Mitochondrial sequence data (590 bp) were prepared for PCA in adegenet 1.4 using the fasta2genlight command; nDNA SNP data were prepared for PCA in adegenet 1.4 using the read.structure command. For all PCAs of molecular data, missing data were replaced using the na.replace command. All analyses described in this section were performed in the R computing environment [51] using the following libraries available from CRAN: adegenet, evobiR, mclust, raster, rgdal, dismo, maptools, and classInt.

### Phylogenetic Analyses

We aligned mtDNA using Sequencher (Gene Codes Corp., Ann Arbor, MI) and MacClade 4 [52]. We conducted phylogenetic analyses using Bayesian [53] and maximum likelihood inference criteria (MEGA 5.1; [54]). For likelihood searches, we assessed nodal support using 2000 bootstrap pseudo-replicates. All tree searches were performed using nearest-neighbor heuristic search criteria and branch swapping. For RADseq data, we used the ‘populations’ module in STACKS 1.09 [55] to identify SNPs that were fixed within individuals but variable among individuals. We used a concatenated alignment of these SNPs (*n* = 2318) to perform a maximum likelihood analysis in MEGA using the GTR+G model of nucleotide evolution and 100 bootstrap pseudoreplicates [56]. We used a maximum of 20% missing data (i.e., 80% of individuals present) as a criterion for selecting SNPs used in RADseq analyses.

### STRUCTURE Analysis of SNP Data

We used 1826 genome-wide SNPs identified using STACKS 1.09 to perform Bayesian clustering as implemented in the program STRUCTURE 2.3 [22]. To identify an appropriate number of clusters we generated BIC scores from *K*-means clustering in adegenet 1.4 [57] and ∆K scores based on five iterations of each *K*-value analyzed by Structure Harvester 0.6.94 [58, 59]. Based on results, we implemented *K*-values of 8–11.

### Tests of Introgression

We tested for introgressive hybridization between well-supported groups using four-taxon Patterson’s D-statistics [24], which test for departures in ancestral allele frequency patterns expected from incomplete lineage sorting. Although use of Patterson’s D-statistics has been found to be unreliable for tests using small genomic regions [60], genome-wide D-statistics are informative for testing statistical excess of shared derived alleles between taxa. We constructed 26 tests to compare allele frequencies from random individuals that represented the four deepest nodes to receive statistical support within the *C*. *mitchellii* complex: (*i*) *C*. *m*. *pyrrhus* mainland populations + *C*. *m*. *muertensis*, (*ii*) *C*. *m*. *mitchellii*, (*iii*) *C*. *m*. *pyrrhus* island populations (minus *C*. *m*. *muertensis*), and (*iv*) *C*. *m*. *angelensis*. We performed multiple tests using either *C*. *stephensi* or *C*. *tigris* as an outgroup taxon (S2 Table). See S2 File for details on the design and implementation of D-statistic tests.

### Species Tree Analysis

We used SNP data to construct a species tree based on our proposed taxonomy using the program SNAPP [61]. We ran one million generations total and sampled at every 1000 generations. To maximize the number of SNPs available for species tree analysis (SNAPP does not allow missing data), we used 26 individuals with large RAD loci catalogs (*C*. *pyrrhus*, 16 individuals; *C*. *mitchellii*, five individuals; *C*. *angelensis*, one individual; *C*. *stephensi*, three individuals; *C*. *tigris*, one individual). Our dataset incorporated 2409 SNPs with 1632 unique patterns.

## Supporting Information

S1 Fig Scatterplots of classification uncertainty for mtDNA (*A*), nDNA (*B*), phenotypic (*C*), and climatic (*D*) data (see Fig 2). The larger and darker the symbol for a given specimen, the higher the classification uncertainty associated with it.
(PDF)

S2 Fig Ordination (PCA) followed by model-based clustering for mitochondrial (*A* and *B*) and nuclear (*C*) datasets. Orange cluster = *Crotalus tigris*, yellow cluster = *C*. *stephensi*, all other clusters represent *C*. *mitchellii* complex (see Fig 2).
(PDF)

S1 File Specimens examined and description of phenotypic characters. (DOCX)

S2 File Design and implementation of Patterson’s D-statistics tests. (DOCX)

S1 Table Factor loadings for the first two axes of a principal components analysis (PCA) of climatic data. The first axis is structured primarily by temperature, and the second by precipitation (see Fig 2D).
(DOCX)

S2 Table Patterson’s D-statistics for select populations and species of the *Crotalus mitchellii* group. The following individuals were used as representatives of focal species and populations: northern representative of *C*. *m*. *mitchellii* (MI1; RWM 1969), southern representative of *C*. *m*. *mitchellii* (MI2; RWM 109), *C*. *m*. *angelensis* (ANG; JMM 225), Mainland *C*. *pyrrhus* from California (PY1; JMM 120), Mainland *C*. *m*. *pyrrhus* from Baja California (PY2; BYU 34764), Mainland *C*. *m*. *pyrrhus* from Arizona (PY3; DGM 834), *C*. *m*. *pyrrhus* from Cabeza de Caballo Island (CCI; JMM 644), *C*. *m*. *pyrrhus* from Piojo Island (PI; JMM 647), *C*. *m*. *pyrrhus* from Smith Island (SI; JMM 654), *C*. *stephensi* (STE; JMM 77), and *C*. *tigris* (TIG; UTEP 18442). Bolded patterns indicate genealogical patterns that are significant and consistent with introgression (*p* ≤ 0.001).
(DOC)

S3 Table Voucher information for specimens sequenced for this study. Country and state abbreviations are as follows: MX = Mexico, USA = United States, BC = Baja California, BCS = Baja California Sur, AZ = Arizona, NV = Nevada, CA = California. Institutional voucher abbreviations are as follows: JMM = Jesse M. Meik field series, DGM = Daniel G. Mulcahy field series, RWM = Robert W. Murphy field series, BTH = Brian T. Hamilton field series, CSL = Carl S. Lieb field series, PCU = Paul C. Ustach field series, TWR = Tod W. Reeder field series, BYU = Brigham Young University, ROM = Royal Ontario Museum, UTA = University of Texas at Arlington. GenBank numbers correspond to sequences that are available online (at http://www.ncbi.nlm.nih.gov/genbank/).
(DOC)
