# Supplementary figures and images for: Limitations of Climatic Data for Inferring Species Boundaries: Insights from Speckled Rattlesnakes
Source: PLoS One. 2015 Jun 24;10(6):e0131435. doi: 10.1371/journal.pone.0131435 (PMC4479545; doi:10.1371/journal.pone.0131435)

A

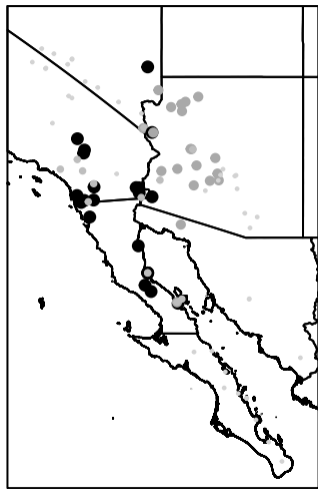

B

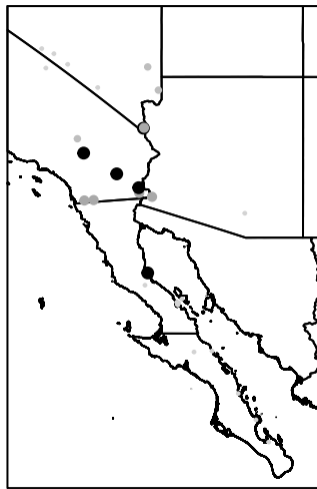

C

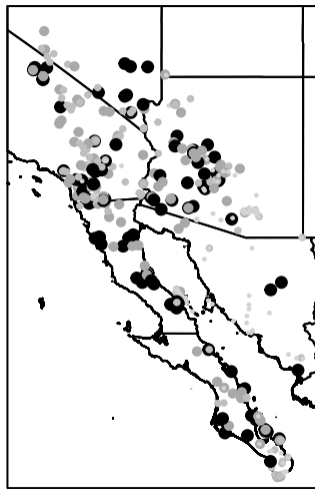

D

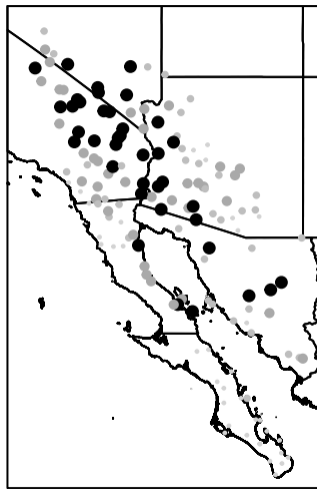

Supplement: S1 Fig — The larger and darker the symbol for a given specimen, the higher the classification uncertainty associated with it. (PDF) [file pone.0131435.s001.pdf]

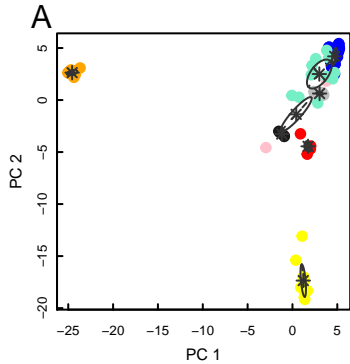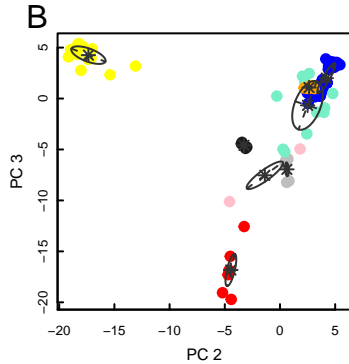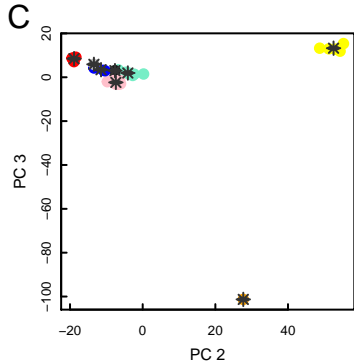

Supplement: S2 Fig — Orange cluster = Crotalus tigris, yellow cluster = C. stephensi, all other clusters represent C. mitchellii complex (see Fig 2). (PDF) [file pone.0131435.s002.pdf]
