# Supplementary material for: Limitations of Climatic Data for Inferring Species Boundaries: Insights from Speckled Rattlesnakes
Source: PLoS One. 2015 Jun 24;10(6):e0131435. doi: 10.1371/journal.pone.0131435 (PMC4479545; doi:10.1371/journal.pone.0131435)
Supplement: S1 File — (DOCX) [file pone.0131435.s003.docx]

**S2 File. Specimens examined and description of phenotypic characters.**

Total specimens examined (*N =*583) from natural history collections. Abbreviations follow Leviton et al. (1) except for the following: MBM = Marjorie Barrick Museum of Natural History, University of Nevada Las Vegas; UABC = Universidad Autónoma de Baja California; MZFC = Museo de Zoología, Facultad de Ciencias, Universidad Nacional Autónoma de México; IBH = Instituto de Biología de Herpetología.

*Crotalus angelensis* (42): BYU 41133; CAS 50869–70, 50904, 103471, 142178; IBH 6152, 6299; MVZ 96803, 96805; MZFC 5012, 5115; SDSNH 19717–18, 19992–95, 44358, 48028–34, 51991–96, 53053; USNM 8562, 15978, 240906, 240908–09. MZFC uncatalogued (3 specimens); UABC uncatalogued (1 specimen).

*Crotalus mitchellii* (96): BYU 34636–37, 34639–40, 34960, 41939; CAS 93, 14030–31, 45887, 52547, 52755, 52816, 52842, 52890, 53711, 101363, 102916, 103470, 146610, 192749, FRM 109, 209, 221, 311, 358, 459, 609, 688; IBH 1045, 1074, 2565, 2605, 3375, 3425; KU 173097; LACM 25083–85, 74029, 104962, 134439; MVZ 11923–24, 13793, 50177, 161442–43, 182176, 190033; MZFC 7597, 16663; SDSNH 2220–22, 2692, 6602–04, 17650–51, 20114, 20498–99, 20563–65, 20780–81, 20784, 20824, 20942, 20946, 21046, 21507, 21596, 22704, 22706, 23096–97, 44359–62, 44675, 45002, 52909, 61226, 68740; UABC 1101, 457; UCM 26136, 26273; USNM 12625, 240364–65, 240691.

*Crotalus pyrrhus* (300): ASU 1606, 2678, 3255, 4585, 9073, 15834, 15836–37, 23276, 24342, 34599, 34607, 34775; BYU 34507–10, 34613, 34667, 34702–03, 35960–61, 46318, 46341, 81355–63; CAS 10108, 17540, 19981–82, 20814, 40091, 90091, 90187, 103466, 143168, 143559, 146566, 146586, 182495, 182499, 182562, 191134, 192750–51, 192754–55, 201174, 214186; IBH 4208; KU 5335, 6996, 31356, 61314, 105920–21, 155535, 174830; LACM 19998, 20006, 28018, 52593–94, 59178, 75838, 104873–74, 104937–38, 104949, 104951, 104960, 112475, 134441–42, 138218–19, 138855; MBM 1929, 1931; MSB 31464, 44451–52, 58361; MVZ 9814, 26661, 41139, 41700, 52012, 52014, 72420, 140884, 161441, 176825, 179937, 193437, 204994–95, 205573, 229800, 229958; SDSNH 534–35, 813, 910, 978, 1067, 2606, 2625, 2778, 2814, 2929–31, 2967–69, 3128–30, 3204, 3297–99, 4304, 4652, 4712, 4732, 4831, 4876, 4930, 4935–36, 5073, 5161, 5570, 7095, 7582–83, 8056, 8557, 8559, 8681, 8703, 8801, 9523, 10023, 10338–39, 11917, 12083, 13366, 16724, 19713–14, 20196, 20596, 20737, 20991, 21093, 22414–15, 23066, 23069, 23149, 23151–54, 23225, 23394–95, 24003, 24103, 24667, 26045, 26090–91, 27537, 27648, 28570, 28734, 29612, 29654, 31776–78, 31835, 31951, 32774, 33777, 34031, 34956, 35005, 35103, 35466, 36336, 36426, 37442–49, 37463–64, 38040, 38995, 39024, 39251, 39815, 39827, 42355, 42970, 44004–09, 44133, 44264, 44359, 44397, 45039, 48027, 49677, 49678–80, 56950–51, 59424, 62291, 63918, 67536, 68862; UABC 1033, 667, 746, 1494–95; UAZ 23296, 27600, 35815–16, 39828, 42974, 42976, 43579, 43580, 43945, 44315, 44801, 45888, 54653–54, 55804; UCM 51221; USNM 16353, 115689, 161166, 205523–24, 222787, 225372–73, 239261, 246618, 248137, 253067–68, 307996, 335504; UTA 7622, 50748, 51392, 51431, 51444, 53198; UTEP 11450, 12147. MZFC uncatalogued (5 specimens); UTA uncatalogued (7 specimens); UABC uncatalogued (4 specimens).

*Crotalus stephensi* (54): BYU 5177–78; CAS 192752, 192757; LACM 36696, 63974, 104931, 134440, 134443; MBM 1959, 3131, 3154, 3160; MVZ 6699, 19344, 228701, 228703; SDSNH 2114, 2970, 4942, 8512, 20986, 21130, 22250, 32519, 32668, 32751, 35117–18, 35122, 35127, 35129, 35131–32, 35141, 35146–47, 35156; TNHC 15329; UAZ 27599; USNM 18662, 18665, 18671–72, 198137, 307994; UTA 51441–42, 53201. UTA uncatalogued (4 specimens).

*Crotalus tigris* (91): ASU 5037, 6690, 6737, 22073, 22272, 22861, 23208, 33150, 33163, 33192, 33224, 33309, 33320; CAS 92265, 103472, 192769; IBH 1724; KU 155525; LACM 25178, 105106–07, 105109–10, 144360; MSB 56030; MVZ 26169, 50841–42, 54622, 70294, 74699, 79234, 206955, 229843, 229845; SDSNH 787, 3132, 3237–38, 3240–43, 17940, 34457, 49715, 49916, 49917; TNHC 30991, 34903; UAZ 27801, 27804, 27807, 27815, 27818–20, 27822, 27824, 27826–27, 27845–48, 33126, 35820, 36516, 36519, 39771, 40084, 41947, 45909, 52169, 53554, 53657; UCM 56930; USNM 80070, 156808, 160399, 238291, 246635; UTA 32248, 54073; UTEP 12324, 18765. UTA uncatalogued (1 specimen).

Phenotypic characters and abbreviations:

1. Number of small scales between the posterior edge of the posterior chin shield and nearest infralabial (POSTG).

2. Number of scales in the prefrontal region between anterior supraoculars and internasals, including canthals (PREFRONT).

3. Number of interrictal scales, counted dorsally from rictus of mouth, excluding ultimate supralabial (RICT).

4. Number of intersupraocular scales (ISUP).

5. Number of interoculabial scales, excluding orbitals and supralabials (IOCUL).

6. Number of scales contacting the rostral scale, including internasals in individuals that do not have nasorostrals (SROSTRAL).

7. Number of foveal scales, including pre-, post-, and subfoveals (FOV).

8. Number of supralabial scales (SUPLAB).

9. Number of infralabial scales (INFLAB).

10. Number of scales contacting the orbit, excluding supraoculars (ORB).

11. Number of scales on the lateral side of the muzzle, including postnasals and loreals (LOREAL).

12. Number of temporal scales, counted as a row that includes two scales above the ultimate supralabial and each scale intervening the second scale and the orbitals (TEMP).

13. Number of ventral scales, including all scutes wider than long, excluding cloacal plate (VENT).

14. Number of subcaudal scales (SUBCAUD).

15. Number of scales fringing the rattle (RFS).

16. Number of middorsal scale rows at center of trunk (MDSR).

17. Number of middorsal scale rows at center of tail (MTDSR).

18. Number of dorsal body rhombs, including parietal blotch (DBB).

19. Number of bands on the tail (TAILBAND).

20. Residual of log_10_-transformed tail length regressed against log_10_-transformed body length (R.RES.TAIL)

21. Residual of log_10_-transformed head length regressed against log_10_-transformed body length (R.RES.HDL)—head length measured from the face of the rostral to the angle of the jaw.

22. Residual of log_10_-transformed head width regressed against log_10_-transformed body length (R.RES.HDW)—head width measured at the widest point, usually just anterior to angle of the jaw.

23. Residual of log_10_-transformed snout length regressed against log_10_-transformed body length (R.RES.SNTL)—snout length measured as distance between anterior edge of orbit and center of rostral.

24. Residual of log_10_-transformed distance between anterior edge of orbit and posterior edge of pit organ regressed against log_10_-transformed body length (R.RES.EYEP).

25. Residual of log_10_-transformed distance between lateral edge of supraocular scales regressed against log_10_-transformed body length (R.RES.ISUP).

26. Residual of log_10_-transformed eye diameter regressed against log_10_-transformed body length (R.RES.EYE).

27. Residual of log_10_-transformed basal rattle segment (end body) height regressed against log_10_-transformed body length (R.RES.BRH).
