# Supplementary material for: Limitations of Climatic Data for Inferring Species Boundaries: Insights from Speckled Rattlesnakes
Source: PLoS One. 2015 Jun 24;10(6):e0131435. doi: 10.1371/journal.pone.0131435 (PMC4479545; doi:10.1371/journal.pone.0131435)
Supplement: S2 File — (DOCX) [file pone.0131435.s004.docx]

**S2 File. Design and interpretation of Patterson’s D-statistic tests.**

We tested for introgressive hybridization between well-supported groups using 26 four-taxon Patterson’s D-statistic tests. We designed these tests to investigate the possibility of introgression explaining some of the inconsistencies between mtDNA, nDNA, and morphological datasets (Fig. 1). In general, we identified fewer shared SNPs between *C. m. mitchellii* and all other taxa. Thus, D-statistic tests involving this taxon were limited to < 1000 unique SNP sites whereas comparisons excluding this species had far more (3296–5041; Table SX). Thus, although we observed some consistent patterns between tests that had < 1000 SNPs and the larger datasets, we acknowledge that increased genomic sampling will be necessary to confidently test for patterns of introgression that involving populations of *C. m. mitchellii*. Because of the sister relationship between *C. stephensi* and *C. tigris* inferred from our species tree analysis (Fig. 4), we performed each test first with *C. stephensi* as the outgroup taxon and second with *C. tigris* in this placement.

The first set of tests was designed to examine for introgression using the topology inferred from our species tree analysis (Fig. 4). Tests 1–4 (Table SX) resulted in non-significant D-statistics.

The second set of tests was designed to test the monophyly of *C. m. mitchellii* populations relative to *C. m. angelensis*. We performed these tests because *C. m. mitchellii* populations clustered separately in both mtDNA and nDNA anlyses (Fig. 1; A–B). We selected *C. m. angelensis* as a candidate for introgression because it was found to have similar PC scores to individuals of *C. m. mitchellii* on several axes in the nDNA PCA (Fig. 1; A). Tests 5-6 (Table x) resulted in non-significant D-statistics which is consistent with the evidence that *C. m. mitchellii* is monophyletic. See also SNP phylogeny (Fig. 3) and morphological clustering (Fig. 1; C).

The third set of tests involved a pattern of topological incongruence between our species tree analysis and mtDNA and nDNA maximum likelihood analyses. Given that our species tree analysis recovered *C. stephensi* and *C. tigris* as sister species (Fig. 4), but our mtDNA and nDNA phylogeny recovered *C. stephensi* as the sister group to the *C. mitchellii* complex, we wanted to investigate the role that introgression may play in generating these differences. Given their parapatric distribution, we used two tests (Tests 7–8; Table x) to investigate the possibility that C. stephensi and C. m. pyrrhus have a history of hybridization. A significant statistic in Test 7 suggests that *C. m. pyrrhus* from California have more SNPs in common with *C. stephensi* than would be expected to result from incomplete lineage sorting. Additionally, although not significant, the proportion of ABBA to BABA sites in Test 8 (involving an individual of *C. m. pyrrhus* from Baja California) is structured in the direction that we be expected if introgression occurred between *C. stephensi* and *C. m. pyrrhus*. We interpret the results as evidence that *C. stephensi* and northern populations of *C. m. pyrrhus* may have a history of periodic hybridization.

The fourth set of tests was designed to test for introgression-like patterns in order to explain a phenomenon in both mtDNA and nDNA datasets: the affinity of several insular and peninsular individuals of *C. m. pyrrhus* and *C. m. angelensis*. We designed multiple tests (Tests 9–26; Table x) that used varying combinations of individuals of *C. m. pyrrhus* which represented populations from Cabeza de Caballo Island, Smith Island, Piojo Island, California, Arizona, and mainland Baja California. These individuals were tested for introgression-like patterns with *C. m. angelensis* (Tests 9–16; Table x) and *C. m. mitchellii* (Tests 17–22; Table x). Most tests including *C. m. pyrrhus* from Baja California (specifically island populations) were significant when gene flow with *C. m. angelensis* was tested (Tests 9–10, 12, 15–16; Table x). Additionally, testing for gene flow between *C. m. mitchellii* and *C. m. pyrrhus* resulted in significant D-statistics when an individual from Cabeza de Caballo island was used (Tests 17–18; Table x). We also tested for patterns consistent with gene flow between northern *C. m. pyrrhus* (originating from California and Arizona) and *C. m. angelensi*s/*C. m. mitchellii* (Tests 23–26; Table x). Only one of these tests returned a significant result (Test 24), and we suspect that this test was significant not because of introgression between *C. m. angelensis* and *C. m. pyrrhus* from Arizona, but rather because *C. stephensi* was used as the outgroup and shares a large number of SNPs with *C. m. pyrrhus* from California (see Test 7). Collectively, we interpret these tests as indicating that complex gene flow dynamics among *C. m. angelensis* and *C. m. pyrrhus* populations inhabiting the Bay of Los Angeles area.
