# Supplementary material for: Limitations of Climatic Data for Inferring Species Boundaries: Insights from Speckled Rattlesnakes
Source: PLoS One. 2015 Jun 24;10(6):e0131435. doi: 10.1371/journal.pone.0131435 (PMC4479545; doi:10.1371/journal.pone.0131435)
Supplement: S1 Table — The first axis is structured primarily by temperature, and the second by precipitation (see Fig 2D). (DOCX) [file pone.0131435.s005.docx]

**S1 Table.** **Factor loadings for the first two axes of a principal components analysis (PCA) of climate data.** The first axis is structured primarily by temperature, and the second by precipitation (see Fig. 2D).

| Variables | PC 1 | PC 2 |
| --- | --- | --- |
| Annual mean temperature | 0.32 | -0.07 |
| Mean diurnal range | -0.08 | -0.07 |
| Isothermality | 0.23 | 0.21 |
| Temperature seasonality | -0.24 | -0.24 |
| Max temperature of warmest month | 0.12 | -0.28 |
| Min temperature of coldest month | 0.34 | 0.04 |
| Temperature annual range | -0.24 | -0.23 |
| Mean temperature of wettest quarter | 0.24 | 0.01 |
| Mean temperature of driest quarter | 0.24 | -0.09 |
| Mean temperature of warmest quarter | 0.23 | -0.21 |
| Mean temperature of coldest quarter | 0.34 | 0.04 |
| Annual precipitation | -0.13 | 0.37 |
| Precipitation of wettest month | -0.001 | 0.41 |
| Precipitation of driest month | -0.29 | 0.07 |
| Precipitation seasonality | 0.26 | 0.27 |
| Precipitation of wettest quarter | -0.03 | 0.41 |
| Precipitation of driest quarter | -0.30 | 0.09 |
| Precipitation of warmest quarter | 0.07 | 0.27 |
| Precipitation of coldest quarter | -0.20 | 0.26 |
