# Supplementary material for: Limitations of Climatic Data for Inferring Species Boundaries: Insights from Speckled Rattlesnakes
Source: PLoS One. 2015 Jun 24;10(6):e0131435. doi: 10.1371/journal.pone.0131435 (PMC4479545; doi:10.1371/journal.pone.0131435)
Supplement: S2 Table — The following individuals were used as representatives of focal species and populations: northern representative of C. m. mitchellii (MI1; RWM 1969), southern representative of C. m. mitchellii (MI2; RWM 109), C. m. angelensis (ANG; JMM 225), Mainland C. pyrrhus from California (PY1; JMM 120), Mainland C. m. pyrrhus from Baja California (PY2; BYU 34764), Mainland C. m. pyrrhus from Arizona (PY3; DGM 834), C. m. pyrrhus from Cabeza de Caballo Island (CCI; JMM 644), C. m. pyrrhus from Piojo Island (PI; JMM 647), C. m. pyrrhus from Smith Island (SI; JMM 654), C. stephensi (STE; JMM 77), and C. tigris (TIG; UTEP 18442). Bolded patterns indicate genealogical patterns that are significant and consistent with introgression (p ≤ 0.001). (DOC) [file pone.0131435.s006.doc]

S2 Table. Patterson’s D-statistics for select populations and species of the *Crotalus mitchellii* group. The following individuals were used as representatives of focal species and populations: northern representative of *C. m. mitchellii* (MI1; RWM 1969), southern representative of *C. m. mitchellii* (MI2; RWM 109), *C. m. angelensis* (ANG; JMM 225), Mainland *C. pyrrhus* from California (PY1; JMM 120), Mainland *C. m. pyrrhus* from Baja California (PY2; BYU 34764), Mainland *C. m. pyrrhus* from Arizona (PY3; DGM 834), *C. m. pyrrhus* from Cabeza de Caballo Island (CCI; JMM 644), *C. m. pyrrhus* from Piojo Island (PI; JMM 647), *C. m. pyrrhus* from Smith Island (SI; JMM 654), *C. stephensi* (STE; JMM 77), and *C. tigris* (TIG; UTEP 18442). Bolded patterns indicate genealogical patterns that are significant and consistent with introgression (p ≤ 0.001).

| **Test topology** | **Sites** | **ABBA** | **BABA** | **D** | **P** | **Putative explanation for significant test result** |
| --- | --- | --- | --- | --- | --- | --- |
|  |  |  |  |  |  |  |
| *Tests based on species tree topology* (Fig. 4) |  |  |  |  |  |  |
|  |  |  |  |  |  |  |
| 1. ((MI1, ANG), PY1), TIG)) | 975 | 18 | 27 | -0.20 | 0.062 | NS |
| 2. ((MI1, ANG), PY1), STE)) | 722 | 11 | 23 | -0.35 | 0.004 | NS |
| 3. ((MI1, ANG), PY2), TIG)) | 723 | 16 | 23 | -0.18 | 0.149 | NS |
| 4. ((MI1, ANG), PY2), STE)) | 704 | 15 | 28 | -0.30 | 0.022 | NS |
|  |  |  |  |  |  |  |
| Test of *C. m. mitchellii* monophyly |  |  |  |  |  |  |
|  |  |  |  |  |  |  |
| 5.((MI1, MI2), ANG), TIG)) | 971 | 11 | 8 | 0.16 | 0.140 | NS |
| 6. ((MI1, MI2), ANG), STE)) | 633 | 10 | 7 | 0.18 | 0.180 | NS |
|  |  |  |  |  |  |  |
| Test of *C. m. angelensis* + *C. m. pyrrhus monophyly* |  |  |  |  |  |  |
|  |  |  |  |  |  |  |
| **7. ((PY1, ANG), STE), TIG))** | **4004** | **47** | **105** | **-0.38** | **0.000** | Introgression between *C. stephensi* and northern *C. m. pyrrhus* |
| 8. ((PY2, ANG), STE), TIG)) | 4082 | 37 | 51 | -0.16 | 0.002 | NS |
|  |  |  |  |  |  |  |
| Tests for introgression between *C. m. angelensis*, *C. m. mitchellii*, and *C. m. pyrrhus* |  |  |  |  |  |  |
|  |  |  |  |  |  |  |
| **9. ((PY2, CCI), ANG), TIG))** | **3867** | **122** | **58** | **0.35** | **0.000** | Introgression between *C. m. angelensis* and insular populations of *C. m. pyrrhus*. |
| **10.** **((PY2, CCI), ANG), STE))** | **5250** | **204** | **127** | **0.23** | **0.000** | Introgression between *C. m. angelensis* and insular populations of *C. m. pyrrhus*. |
| 11. ((PY2, PI), ANG), TIG)) | 3955 | 100 | 75 | 0.14 | 0.004 | NS |
| **12. ((PY2, PI), ANG), STE))** | **5041** | **165** | **112** | **0.19** | **0.000** | Introgression between *C. m. angelensis* and insular populations of *C. m. pyrrhus*. |
| 13. ((PY2, SI), ANG), TIG)) | 3296 | 81 | 63 | 0.13 | 0.036 | NS |
| 14. ((PY2, SI), ANG), STE)) | 4460 | 136 | 107 | 0.12 | 0.017 | NS |
| **15. ((PY1, PY2), ANG), TIG))** | **3810** | **178** | **57** | **0.52** | **0.000** | Introgression between *C. m. angelensis* and insular populations of *C. m. pyrrhus*. |
| **16. ((PY1, PY2), ANG), STE))** | **4029** | **354** | **67** | **0.46** | **0.001** | Introgression between *C. m. angelensis* and mid-peninsula populations of *C. m. pyrrhus*. |
| **17. ((PY2, CCI)), MI1), TIG))** | **581** | **19** | **7** | **0.46** | **0.001** | Shared ancestral alleles between *C. m. angelensis* and *C. m. mitchellii* are found in CCI individuals of *C. m. pyrrhus* as a result of introgression with *C. m. angelensis* (Tests 9–10). |
| **18. ((PY2, CCI)), MI1), STE))** | **595** | **34** | **6** | **0.70** | **0.000** | Shared ancestral alleles between *C. m. angelensis* and *C. m. mitchellii* are found in CCI individuals of *C. m. pyrrhus* as a result of introgression with *C. m. angelensis* (Tests 9–10). |
| 19. ((PY2, PI)), MI1), TIG)) | 620 | 9 | 8 | 0.06 | 0.665 | NS |
| 20. ((PY2, PI)), MI1), STE)) | 627 | 15 | 8 | 0.30 | 0.029 | NS |
| 21.((PY2, SI)), MI1), TIG)) | 471 | 10 | 6 | 0.25 | 0.108 | NS |
| 22. ((PY2, SI)), MI1), STE)) | 498 | 17 | 9 | 0.31 | 0.048 | NS |
| 23. ((PY1, PY3)), ANG), TIG)) | 3972 | 61 | 47 | 0.13 | 0.012 | NS |
| **24. ((PY1, PY3)), ANG), STE))** | **4058** | **127** | **62** | **0.34** | **0.000** | Introgression between *C. stephensi* and northern *C. m. pyrrhus* generated higher number of “A” sites between these taxa |
| 25. ((PY1, PY3)), MI1), TIG)) | 796 | 10 | 7 | 0.18 | 0.130 | NS |
| 26. ((PY1, PY3)), MI1), STE)) | 610 | 14 | 7 | 0.33 | 0.013 | NS |
|  |  |  |  |  |  |  |
