# Supplementary material for: Limitations of Climatic Data for Inferring Species Boundaries: Insights from Speckled Rattlesnakes
Source: PLoS One. 2015 Jun 24;10(6):e0131435. doi: 10.1371/journal.pone.0131435 (PMC4479545; doi:10.1371/journal.pone.0131435)
Supplement: S3 Table — Country and state abbreviations are as follows: MX = Mexico, USA = United States, BC = Baja California, BCS = Baja California Sur, AZ = Arizona, NV = Nevada, CA = California. Institutional voucher abbreviations are as follows: JMM = Jesse M. Meik field series, DGM = Daniel G. Mulcahy field series, RWM = Robert W. Murphy field series, BTH = Brian T. Hamilton field series, CSL = Carl S. Lieb field series, PCU = Paul C. Ustach field series, TWR = Tod W. Reeder field series, BYU = Brigham Young University, ROM = Royal Ontario Museum, UTA = University of Texas at Arlington. GenBank numbers correspond to sequences that are available online (at http://www.ncbi.nlm.nih.gov/genbank/). (DOC) [file pone.0131435.s007.doc]

S3 Table. Voucher information for specimens sequenced for this study. Country and state abbreviations are as follows: MX = Mexico, USA = United States, BC = Baja California, BCS = Baja California Sur, AZ = Arizona, NV = Nevada, CA = California. Institutional voucher abbreviations are as follows: JMM = Jesse M. Meik field series, DGM = Daniel G. Mulcahy field series, RWM = Robert W. Murphy field series, BTH = Brian T. Hamilton field series, CSL = Carl S. Lieb field series, PCU = Paul C. Ustach field series, TWR = Tod W. Reeder field series, BYU = Brigham Young University, ROM = Royal Ontario Museum, UTA = University of Texas at Arlington. GenBank numbers will be provided upon acceptance.

| **Species** | **Voucher** | **Country, State** | **Locality** | **GPS** | **ATPase 6 and 8** | **No. RAD loci** |
| --- | --- | --- | --- | --- | --- | --- |
|  |  |  |  |  |  |  |
| *Crotalus angelensis* | JMM 225 | MX, BC | Angel de la Guarda | -113.332591, 29.166829 | TBD | 3725 |
| *Crotalus angelensis* | JMM 226 | MX, BC | Angel de la Guarda | -113.332591, 29.166829 | TBD | N/A |
| *Crotalus mitchellii* | BYU 34636 | MX, BCS | Isla Monserrate | -111.042791, 25.678914 | TBD | N/A |
| *Crotalus mitchellii* | BYU 34640 | MX, BCS | Isla Monserrate | -111.037053, 25.666109 | TBD | N/A |
| *Crotalus mitchellii* | ROM 35409 | MX, BCS | San Evaristo (village on coast opposite Isla San Jose) | -110.711284, 24.907256 | TBD | 674 |
| *Crotalus mitchellii* | RWM 1969 | MX, BCS | La Paz | -110.260063, 24.124551 | TBD | 940 |
| *Crotalus mitchellii* | RWM 922 | MX, BCS | San Jose de Comondu | -111.836005, 26.033321 | TBD | N/A |
| *Crotalus mitchellii* | BYU 34748 | MX, BCS | Juncalito | -111.338018, 25.834791 | TBD | 3534 |
| *Crotalus mitchellii* | JRO 488 | MX, BCS | San Ignacio | -112.901683, 27.300456 | TBD | 1887 |
| *Crotalus mitchellii* | RWM 109 | MX, BCS | Santa Rosalia | -112.267138, 27.333418 | TBD | 3132 |
| *Crotalus pyrrhus* | JMM 643 | MX, BC | Cabeza de Caballo | -113.478753, 28.972609 | TBD | 3754 |
| *Crotalus pyrrhus* | JMM 644 | MX, BC | Cabeza de Caballo | -113.478753, 28.972609 | TBD | 3767 |
| *Crotalus pyrrhus* | JMM 645 | MX, BC | Cabeza de Caballo | -113.478753, 28.972609 | TBD | 3777 |
| *Crotalus pyrrhus* | JMM 646 | MX, BC | Cabeza de Caballo | -113.478753, 28.972609 | TBD | 3772 |
| *Crotalus pyrrhus* | JMM 642 | MX, BC | Cabeza de Caballo | -113.478753, 28.972609 | TBD | 3738 |
| *Crotalus pyrrhus* | DGM 834 | USA, AZ | Yuma Co., hwy 95, near Castle Dome Mtns | -114.386667, 32.773167 | TBD | 3764 |
| *Crotalus pyrrhus* | BTH 518 | USA, NV | Lincoln Co., Rainbow Canyon | -114.54, 37.354 | TBD | 3746 |
| *Crotalus pyrrhus* | BYU 34508 | MX, BC | San Felipe | -114.872433, 31.042623 | TBD | N/A |
| *Crotalus pyrrhus* | BYU 34764 | MX, BC | Santa Inez | -114.6352, 29.655273 | TBD | 3581 |
| *Crotalus pyrrhus* | CSL 8840 | USA, CA | San Bernardino Co., San Bernardino Mtns | -116.805, 34.315 | TBD | 3627 |
| *Crotalus pyrrhus* | Dulzura | USA, CA | San Diego Co., Dulzura (Ben Lowe) | -116.780662, 32.644123 | TBD | 3296 |
| *Crotalus pyrrhus* | Gold Butte | USA, NV | Clark Co., Gold Butte, Virgin Mtns | -114.161663, 36.53489 | TBD | 3758 |
| *Crotalus pyrrhus* | JMM 120 | USA, CA | San Bernardino Co., S of Barstow | -117.026099, 34.821381 | TBD | 3695 |
| *Crotalus pyrrhus* | JMM 140 | USA, AZ | Yavapai Co., near Bagdad | -112.993272, 34.454002 | TBD | N/A |
| *Crotalus pyrrhus* | JMM 171 | MX, BC | Smith | -113.50443, 29.052182 | TBD | N/A |
| *Crotalus pyrrhus* | JMM 220 | MX, BC | El Muerto | -114.540779, 30.08781 | TBD | 1383 |
| *Crotalus pyrrhus* | JMM 221 | MX, BC | El Muerto | -114.540779, 30.08781 | TBD | 3364 |
| *Crotalus pyrrhus* | JMM 641 | MX, BC | Cabeza de Caballo | -113.478753, 28.972609 | TBD | 3396 |
| *Crotalus pyrrhus* | JMM 647 | MX, BC | El Piojo | -113.464008, 29.01728 | TBD | 3706 |
| *Crotalus pyrrhus* | JMM 648 | MX, BC | El Piojo | -113.464008, 29.01728 | TBD | 3646 |
| *Crotalus pyrrhus* | JMM 649 | MX, BC | El Piojo | -113.464008, 29.01728 | TBD | 3689 |
| *Crotalus pyrrhus* | JMM 650 | MX, BC | El Piojo | -113.464008, 29.01728 | TBD | 3775 |
| *Crotalus pyrrhus* | JMM 651 | MX, BC | El Piojo | -113.464008, 29.01728 | TBD | 3750 |
| *Crotalus pyrrhus* | JMM 652 | MX, BC | Smith | -113.50443, 29.052182 | TBD | 3742 |
| *Crotalus pyrrhus* | JMM 653 | MX, BC | Smith | -113.50443, 29.052182 | TBD | 3649 |
| *Crotalus pyrrhus* | JMM 654 | MX, BC | Smith | -113.50443, 29.052182 | TBD | 3631 |
| *Crotalus pyrrhus* | UTA R-53198 | USA, CA | Imperial Co., Ogilby Rd | -114.839808, 32.909554 | TBD | 3692 |
| *Crotalus pyrrhus* | PCU 565 | USA, NV | Clark Co., Newberry Mtns | -114.692899, 35.193191 | TBD | 3588 |
| *Crotalus pyrrhus* | PCU 575 | USA, NV | Clark Co., Newberry Mtns | -114.670098, 35.207009 | TBD | 3570 |
| *Crotalus pyrrhus* | ROM 13522 | MX, BC | Catavina | -114.420866, 29.43248 | TBD | N/A |
| *Crotalus pyrrhus* | ROM 38475 | MX, BC | El Muerto | -114.540779, 30.08781 | TBD | N/A |
| *Crotalus pyrrhus* | ROM 38478 | MX, BC | El Muerto | -114.540779, 30.08781 | TBD | N/A |
| *Crotalus pyrrhus* | RWM 1813 | MX, BC | Bahia Los Angeles | -113.565612, 28.947092 | TBD | N/A |
| *Crotalus pyrrhus* | TWR 1142 | USA, CA | Riverside Co. | -115.636, 33.582 | TBD | 3327 |
| *Crotalus pyrrhus* | TWR 2672 | USA, CA | San Diego Co. | -116.894, 32.575 | TBD | N/A |
| *Crotalus pyrrhus* | TWR 2676 | USA, CA | San Diego Co., La Posta | -116.4527, 32.6581 | TBD | 2844 |
| *Crotalus pyrrhus* | TWR 389 | USA, CA | San Diego Co., Hwy 78 w of Anzo Borrego | -116.437, 33.119 | TBD | N/A |
| *Crotalus pyrrhus* | TWR 903 | USA, CA | Imperial Co., Chocolate Mountains | -114.949, 33.099, | TBD | N/A |
| *Crotalus pyrrhus* | UTEP 17628 | USA, CA | Imperial Co., Black Mountain Rd, 3.3 mi SE jct with Sthwy 78 | -114.859375, 33.094462 | TBD | 3593 |
| *Crotalus stephensi* | BTH 38 | USA, NV | Esmeralda Co., Coyote road, Silver Peak Range, 7.7 miles east of the crossing | -114.859375, 33.094462 | TBD | 3628 |
| *Crotalus stephensi* | BTH 39 | USA, NV | Mineral Co., Just off Hwy. 6, 0.9 miles west of Hwy. 360 | -117.855, 37.82 | TBD | 3555 |
| *Crotalus stephensi* | JMM 77 | USA, CA | Inyo Co., Inyo Range | -118.139, 37.3137 | TBD | 3575 |
| *Crotalus stephensi* | PCU 591 | USA, NV | Nye Co., Speckter Range | -116.305614, 36.627325 | TBD | 3648 |
| *Crotalus stephensi* | PCU 648 | USA, NV | Esmeralda Co. | -117.367, 37.446 | TBD | 3320 |
| *Crotalus tigris* | KWS 252 | USA, AZ | Pima Co., Collosus Cave | -110.667778, 32.060594 | TBD | N/A |
| *Crotalus tigris* | UTEP 18442 | USA, AZ | Pima Co., Foothills of Golden Gate Mtn, Dor Kinney Rd | -111.105202, 32.193154 | TBD | 3226 |
|  |  |  |  |  |  |  |
